# Cu_2_ZnSnS_4_/MoS_2_-Reduced Graphene Oxide Heterostructure: Nanoscale Interfacial Contact and Enhanced Photocatalytic Hydrogen Generation

**DOI:** 10.1038/srep39411

**Published:** 2017-01-03

**Authors:** Enna Ha, Wei Liu, Luyang Wang, Ho-Wing Man, Liangsheng Hu, Shik Chi Edman Tsang, Chris Tsz-Leung Chan, Wai-Ming Kwok, Lawrence Yoon Suk Lee, Kwok-Yin Wong

**Affiliations:** 1 Department of Applied Biology and Chemical Technology and the State Key Laboratory of Chirosciences, The Hong Kong Polytechnic University, Hung Hom, Kowloon, Hong Kong SAR, China; 2 Wolfson Catalysis Centre, Department of Chemistry, University of Oxford, Oxford, OX1 3QR, UK

## Abstract

Hydrogen generation from water using noble metal-free photocatalysts presents a promising platform for renewable and sustainable energy. Copper-based chalcogenides of earth-abundant elements, especially Cu_2_ZnSnS_4_ (CZTS), have recently arisen as a low-cost and environment-friendly material for photovoltaics and photocatalysis. Herein, we report a new heterostructure consisting of CZTS nanoparticles anchored onto a MoS_2_-reduced graphene oxide (rGO) hybrid. Using a facile two-step method, CZTS nanoparticles were *in situ* grown on the surface of MoS_2_-rGO hybrid, which generated high density of nanoscale interfacial contact between CZTS and MoS_2_-rGO hybrid. The photoexcited electrons of CZTS can be readily transported to MoS_2_ through rGO backbone, reducing the electron-hole pair recombination. In photocatalytic hydrogen generation under visible light irradiation, the presence of MoS_2_-rGO hybrids enhanced the hydrogen production rate of CZTS by 320%, which can be attributed to the synergetic effect of increased charge separation by rGO and more catalytically active sites from MoS_2_. Furthermore, this CZTS/MoS_2_-rGO heterostructure showed much higher photocatalytic activity than both Au and Pt nanoparticle-decorated CZTS (Au/CZTS and Pt/CZTS) photocatalysts, indicating the MoS_2_-rGO hybrid is a better co-catalyst for photocatalytic hydrogen generation than the precious metal. The CZTS/MoS_2_-rGO system also demonstrated stable photocatalytic activity for a continuous 20 h reaction.

Semiconductor nanomaterials possess several useful intrinsic properties, such as morphology-dependent optical properties, capability of converting solar energy into either electrical or chemical energy, and large surface area-to-volume ratio, which have triggered tremendous research efforts in developing new class of materials in the last decade, especially in the fields of photocatalysis^1^,^2^,^3^,^4^, solar cells^5^,^6^, and light emitting devices^7^. Oxide semiconductors, which have been widely used as a light absorbing material, usually have a wide band gap due to the low lying valence band (VB) composed of oxygen orbitals^8^,^9^,^10^,^11^,^12^. This poses a limit on the useful absorption range for exciton generation and thus lowers the power conversion efficiency in many cases of oxides. Sulfide materials, on the other hand, possess narrower band gaps than oxides, and have recently gained much research interests as alternatives for oxides^13^,^14^,^15^. Particularly, a quaternary copper-based chalcogenide Cu_2_ZnSnS_4_ (CZTS) has shown a number of advantages in harvesting and harnessing the solar energy. The low band gap of around 1.5 eV makes CZTS a suitable light absorbing material for both ultra-violet (UV) and visible lights. Moreover, all the components in CZTS are earth abundant, low-cost, and less-toxic, making it a sustainable clean energy converter^16^,^17^,^18^. In addition to its application as a light absorber in photovoltaic devices, CZTS has been also engaged in the photocatalytic hydrogen generation as an effective catalyst and demonstrated better catalytic performance and stability than commercialized CdS and N-doped TiO_2_ for visible light hydrogen production^19^. Precious metals with high catalytic activity and surface plasmon resonance effect, such as Pt and Au, have been decorated on or incorporated with CZTS as a co-catalyst, which resulted in a significant enhancement in photocatalytic hydrogen production^20^,^21^,^22^. However, the scarcity and high cost of these noble metals void their competitiveness and hamper their widespread use in industry. It would be much advantageous if the catalytic efficiency of CZTS can be improved by coupling with other low cost but high performance materials through a delicate interfacial design.

Constructing heterostructure stands out as an effective way to transfer the excitons away from the recombination sites and increase the number of active sites for catalytic processes^23^,^24^,^25^. Moreover, nanoscale interfacial contact in the heterostructures has also been proved as a fast and shorter channel for exciton transportation, which further inhibits the recombination^26^,^27^,^28^. The nanoscale interfacial contact can be formed through epitaxial growth, where one composite grows on the other crystalline substrate through the same structural orientation^29^. In the NaTaO_3_/Ta_2_O_5_ heterostructure, NaTaO_3_ was grown epitaxially along [121] direction on the (110) Ta_2_O_5_ crystal plane^30^. Compared to the single components, the photocatalytic hydrogen generation yield of this heterostructure has more than doubled. It was also reported that the heterostructure of CdS and ZnIn_2_S_4_ with mismatched lattice formed a nanointerface through dangling bonds^31^. The resulting helical CdS/ZnIn_2_S_4_ has demonstrated three-fold enhancement in the photocurrent intensity. These superior properties arisen from nanoscale interfacial contact have inspired us to develop the CZTS-based heterostructures.

Two dimensional materials, especially graphene and MoS_2_, possess unique properties of large surface area, fast electron mobility, and superior catalytic ability, all of which make them highly attractive for numerous applications^32^,^33^,^34^,^35^,^36^,^37^. In the study of Bi_2_WO_6_-MoS_2_-reduced graphene oxide (rGO) heterostructure, MoS_2_-rGO hybrid has shown the confined space effect for the synthesis of Bi_2_WO_6_ and the nanoscale interfacial contact results in the enhanced photocatalytic properties. By coupling rGO and MoS_2_ with CZTS into a robust heterostructure with nanoscale interfacial contacts, we expect pronounced enhancements in photocatalytic performance of CZTS, which is currently not as competitive as other systems. Herein, we report a simple method of incorporating CZTS nanoparticles with rGO, few-layer MoS_2_, and MoS_2_-rGO hybrids, and their photocatalytic performances in hydrogen generation under visible light irradiation. We found that the photocatalytic activity of CZTS/MoS_2_-rGO heterostructure outperforms those of many other hydrogen photocatalysts based on earth abundant elements reported in the literature, and it also showed excellent long-term stability^38^,^39^,^40^.

## Results and Discussion

**Figure 1:**
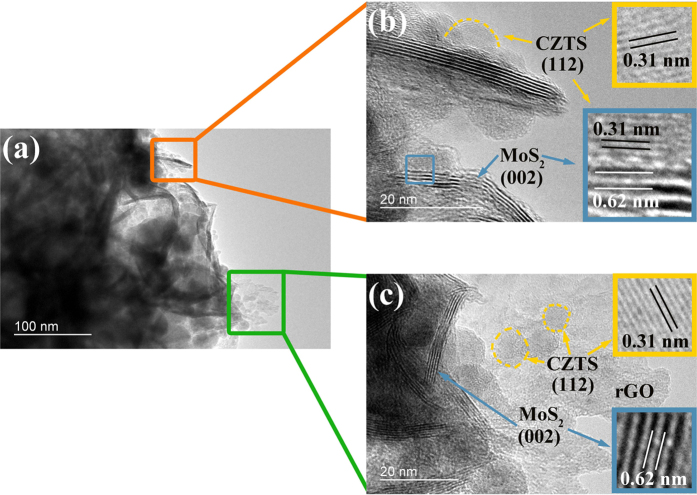
Typical (a) TEM and (b), (c) high-resolution TEM images of as-prepared CZTS/MoS_2_-rGO composites.

The CZTS/MoS_2_-rGO heterostructure was prepared by a two-step reaction. In the first step, MoS_2_-rGO hybrid was synthesized by hydrothermal reaction where few-layered MoS_2_ nanosheets were constructed in the presence of graphene oxide. In the following colloidal synthesis, Cu-Zn-Sn-oleylamine precursors and sulfur source were nucleated and grown to nanoparticles in the presence of MoS_2_-rGO hybrids. Figure 1 displays typical transmission electron microscopic (TEM) images of as-synthesized CZTS/MoS_2_-rGO heterostructure where spherical CZTS nanoparticles were in nanoscale interfacial contact with MoS_2_-rGO hybrid substrates. High-resolution TEM images of CZTS/MoS_2_-rGO heterostructure, shown in Fig. 1(b) and (c), clearly reveal the lattice fringes from each component and the interface, indicating their high degree of crystallinity and close contact. The CZTS nanoparticles are slightly polydispersed with most of the nanoparticle sizes falling in the range of 5–20 nm (Fig. S1 in the Supporting Information). The average particle size is 10.5 ± 2.7 nm. MoS_2_ displays a multi-layered structure with an interlayer spacing of 0.62 nm which corresponds to the (002) planes of hexagonal MoS_2_. The measured interplanar d-spacing of 0.32 nm in the light-colored area can be ascribed to the (002) plane of kesterite CZTS. In the synthetic procedure, MoS_2_-rGO hybrid and sulfur were dispersed in oleylamine and sonicated. It is well known that sulfur reacts with oleylamine and produces alkylammonium polysulfides during the sonication^41^. At the same time, the sulfur ions bind to the exposed Mo atoms of MoS_2_ and defective sites of rGO through covalent binding^42^. Upon heating at high temperature, H_2_S is *in situ* produced and reacts with the metal precursors to form CZTS on the surface of MoS_2_-rGO hybrid^43^. The co-existence of four constituent elements in CZTS as well as Mo was confirmed by energy dispersive spectroscopy (EDS) and elemental mapping (Figs S2 and S3). TEM images of CZTS, MoS_2_, GO, CZTS/MoS_2_, CZTS/rGO, and MoS_2_-rGO hybrids were also taken and shown in Fig. S4 for comparison.

**Figure 2:**
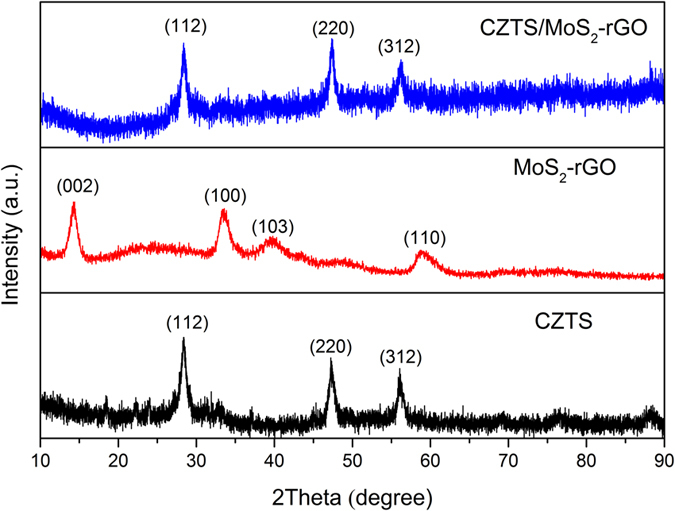
Powder XRD patterns of as-synthesized CZTS, MoS_2_-rGO, and CZTS/MoS_2_-rGO composite.

X-ray powder diffraction (XRD) patterns obtained from as-synthesized MoS_2_-rGO, CZTS, and CZTS/MoS_2_-rGO are shown in Fig. 2. XRD results confirmed that the presence of MoS_2_ and rGO did not alter the crystal phase of CZTS nor produce any binary/tertiary byproducts. All the diffraction peaks of the heterostructure are well indexed according to the kesterite phase of CZTS. There was, however, no characteristic diffraction peaks for MoS_2_ and carbon species observed because of the small weight ratio in the heterostructure (10 wt%) and relatively low diffraction intensity of MoS_2_ and rGO^44^,^45^. X-ray photoelectron spectroscopy (XPS) analyses were performed to investigate the surface elements and chemical states of CZTS/MoS_2_-rGO heterostructure. The XPS survey spectrum shown in Fig. S5(a) identified the presence of Cu, Zn, Sn, S, Mo, and C in the CZTS/MoS_2_-rGO heterostructure. In the high resolution XPS spectrum of graphene oxide (Fig. S5(b)), C-O and C-C peaks appear with strong intensities and O=C–O peak as a shoulder at 286.2, 284.2, and 288.0 eV, respectively. The CZTS/MoS_2_-rGO heterostructure showed a major C 1 s signal from C-C at 284.9 eV and a tiny C-O peak at 286.1 eV (Fig. S5(c)). The O=C–O peak has completely disappeared and C-O intensity has significantly decreased, indicating the full reduction of graphene oxide to rGO and reconstruction of π-conjugation in rGO plane during the synthesis of CZTS/MoS_2_-rGO heterostructure^44^,^46^,^47^. Two peaks observed at 933.2 and 953.2 eV in the high-resolution XPS spectra were determined to be 2p_3/2_ and 2p_1/2_ states of Cu(I). Zn(II) state was identified from the peak at 1,022.6 eV. The sharp Sn 3d peaks located at 486.9 and 495.4 eV with a peak separation of 8.5 eV confirmed the Sn(IV) state. The sulfur 2p_3/2_ and 2p_1/2_ peaks were identified at 162.4 and 163.4 eV, which agreed with the sulfide phase in the range of 160 and 164 eV. The Mo 3d spectrum showed two peaks at 228.9 and 232 eV which were attributed to the 3d_3/2_ and 3d_5/2_ orbitals, respectively.

**Figure 3:**
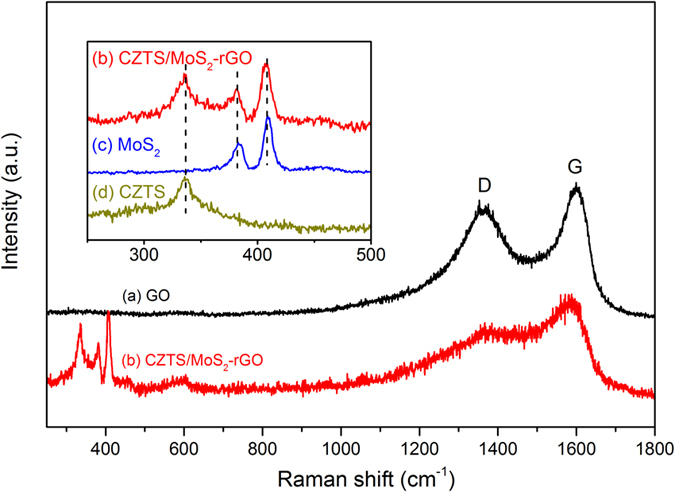
Raman spectra of as-synthesized (**a**) GO and (**b**) CZTS/MoS_2_-rGO composite. Inset shows the comparison of Raman spectra of (**b**) CZTS/MoS_2_-rGO, (**c**) MoS_2_, and (**d**) CZTS in the region of 250 to 500 cm^−1^.

In order to further ensure the structural homogeneity, Raman measurements were taken and are shown in Fig. 3. Two peaks at 1,596 and 1,360 cm^−1^, observed from a pre-synthesized GO, correspond to G and D bands. The G band is related to the in-plane vibration mode of sp^2^ carbon, while D band demonstrates defective and partially disordered structures of the carbon to sp^3^ hybrid carbon. All the characteristic Raman bands for CZTS and few-layer MoS_2_ can be found at 335, 383, and 409 cm^−1^, corresponding to the 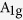 mode of CZTS and 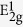, 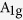 modes of MoS_2_, respectively^43^,^48^,^49^. The Raman spectrum of CZTS/MoS_2_-rGO heterostructure also reveals the coexistence of all three components with high purity. The heating in oleylamine at high temperature (280 °C) is believed to induce the reduction of graphene oxide to rGO^43^. The optical properties of heterostructure are also affected by the formation of nanoscale interfacial contact between two different materials. The UV-Vis spectra obtained from CZTS/MoS_2_-rGO heterostructure, pristine CZTS nanoparticles, and MoS_2_-rGO hybrids are compared in Fig. S6. The pristine CZTS nanoparticle displays a band edge of around 1.3 eV which matches well with the literature value^17^. Three distinct peaks observed from MoS_2_-rGO at *ca*. 460, 620, and 665 nm originate from the band gap transition from the valence band to the conduction band of MoS_2_^50^. The UV-Vis spectrum of CZTS/MoS_2_-rGO heterostructure is similar to that of CZTS, but a small shoulder at *ca*. 460 nm and a broad peak at 600–850 nm indicate that MoS_2_-rGO has been successfully incorporated with CZTS.

**Figure 4:**
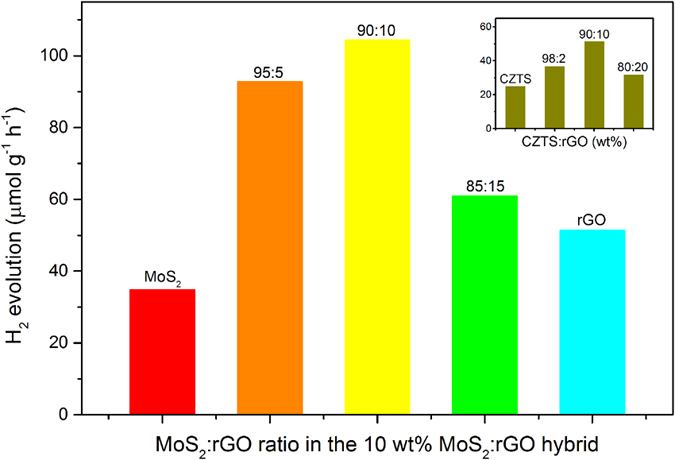
Comparison of photocatalytic H_2_ evolution from various CZTS/MoS_2_-rGO composites with different ratios of MoS_2_-rGO co-catalyst. All samples contain 10 wt% co-catalyst incorporated with 90 wt% CZTS. Inset: comparison of photocatalytic H_2_ evolution from CZTS/rGO composites of various ratios. Experiment conditions: 1 h irradiation by solar simulator (150 W Xe lamp).

Comparative experiments on the photocatalytic H_2_ evolution were conducted using CZTS nanoparticle and its heterostructure with MoS_2_ and/or rGO. Figure 4 summarizes the results of the H_2_ evolution yields from CZTS/MoS_2_-rGO heterostructure (5 mg sample size) under 1 h illumination of simulated sunlight. CZTS nanoparticle alone was photocatalytically active, but the rate of H_2_ evolution was low (25 μmol g^−1^ h^−1^, inset in Fig. 4). We first prepared CZTS/rGO heterostructure with different rGO wt% to study how the presence of rGO affects the rate of H_2_ production (inset in Fig. 4). The addition of 2 wt% rGO to CZTS slightly enhanced H_2_ generation rate to 37 μmol g^−1^ h^−1^. With rGO ratio of 10 wt%, the photocatalytic yield was more than doubled (52 μmol g^−1^ h^−1^) which is 106% enhancement. However, further increase of rGO content decreased H_2_ production due to the shading effect of rGO that could block the active sites of CZTS^23^. Accordingly, we have fixed the amount of hybrid cocatalyst to 10 wt% for the subsequent experiments where both rGO and MoS_2_ were engaged. The enhancement effect from the MoS_2_-rGO hybrid was much more profound than when rGO or MoS_2_ was used as a single component. When 10 wt% MoS_2_-rGO hybrid with 95:5 MoS_2_: rGO ratio was incorporated into CZTS, the H_2_ evolution yield was enhanced by more than 270%. A slight increase of rGO ratio in the MoS_2_-rGO hybrid to 90:10 further enhanced the rate of H_2_ evolution to 320%. However, the photocatalytic activity of the heterostructure decreased with more than 1 wt% rGO content. Incorporation of 10 wt% MoS_2_ as a single component resulted in merely 40% enhancement in H_2_ production. In a series of control experiments, the photocatalytic H_2_ evolution from GO, MoS_2_, MoS_2_-rGO hybrid, Au nanoparticle decorated CZTS (Au/CZTS) and Pt nanoparticle decorated CZTS (Pt/CZTS) were also tested for comparison (Figs S7 and S8). All of these systems showed much lower photocatalytic activities than CZTS/MoS_2_-rGO heterostructure. Bare MoS_2_ and MoS_2_-rGO showed low photocatalytic activities for H_2_ generation, due to the intrinsic indirect band gap that favors a rapid non-radiative relaxation in the form of phonons (heat) and increases the probability for electron-hole pair recombination^46^,^50^. It is worthwhile to note that the H_2_ yields from both Au/CZTS and Pt/CZTS are less than half of that from CZTS/MoS_2_-rGO heterostucture, demonstrating that a rational design of low-cost nanomaterial heterostucture outperforms the noble metal-loaded semiconductor catalysts. The stability of CZTS/MoS_2_-rGO heterostructure was investigated by a prolonged 20 h reaction (Fig. S9). The H_2_ yield from CZTS/MoS_2_-rGO heterostructure showed a linear correlation with reaction time, indicating its high photocatalytic stabilities under UV-Vis light.

**Figure 5:**
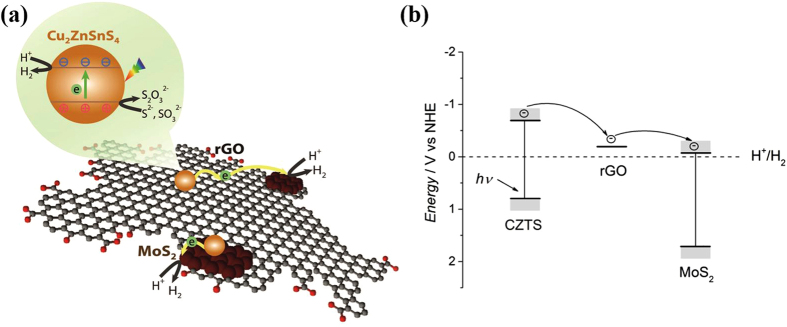
Illustration and energy diagram of interfacial charge transfer and photocatalytic redox reaction in CZTS/MoS_2_-rGO composites (the band values are taken from references 20 and 44).

We also carried out a series of electrocatalytic H_2_ evolution tests in a typical three-electrode setup using a platinum wire and a saturated calomel electrode (SCE) as the counter and the reference electrode, respectively (Fig. S10). An alkaline solution containing Na_2_S (0.35 M) + Na_2_SO_3_ (0.25 M) was used as the electrolyte in order to maintain the same conditions as in the photocatalytic experiments for comparison. The photocatalyst samples (5 mg) containing various ratios of CZTS, MoS_2_, and rGO have been dispersed in Nafion solution and deposited onto a glassy carbon electrode (GCE) by drop-casting. Bare GCE was used as a background reference system. The polarization curve recorded with CZTS-modified GCE showed a poor catalytic activity with the onset of H_2_ evolution at −1.62 V. The CZTS (90 wt%)/rGO (10 wt%)-modified GCE exhibited a slight enhancement in cathodic current and reduction of overpotential. When 10 wt% MoS_2_ was used with CZTS instead of rGO, further reduction in the onset potential was observed. MoS_2_, which has larger size than CZTS, could also provide efficient electron transfer path, as well as additional active sites for H_2_ generation. With both MoS_2_ (9 wt%) and rGO (1 wt%) present in the heterostructure, the highest electrocatalytic current density and lowest onset potential were obtained. As suggested by the TEM images (Fig. 1), CZTS, MoS_2_, and rGO are in nanoscale contacts with one another and this leads to a much faster electron transfer across the entire heterostructure. The presence of highly conductive rGO carbon network^51^ coupled with the superior electrocatalytic activity from the edge of MoS_2_ synergistically enhanced the activity of CZTS/MoS_2_-rGO heterostructure for electrochemical water reduction^44^,^52^. The introduction of MoS_2_-rGO hybrid into the CZTS can also increase the number of active sites for both photocatalytic and electrocatalytic H_2_ production applications. A proposed photocatalytic H_2_ production mechanism is as follows (Fig. 5). Upon the absorption of light, CZTS nanoparticles generate excited electrons and holes. The comparable energy differences among the conduction band of CZTS, Fermi level of rGO, and conduction band of MoS_2_ allow the photoexcited electrons to be either directly transferred to the nearby MoS_2_ or shuttled across rGO backbone to a remote MoS_2_, and consumed for H_2_ reduction. It is not yet known how the lifetime of excitons in CZTS is modified in the presence of MoS_2_-rGO, but the rapid transfer and isolation of excited electrons from the immobile holes of CZTS to MoS_2_ *via* rGO will certainly reduce the radiative recombination of photogenerated electron-hole pairs and prolong the exciton lifetime^37^,^46^,^53^,^54^. The produced photoelectrons on the edge sulfide sites (both CZTS and MoS_2_) are captured by H^+^ and used to reduce H^+^ to H_2_, while the holes are consumed by sacrificial agents, S^2−^ and SO_3_^2−^, to complete the catalytic cycle.

In conclusion, we have shown the fabrication of CZTS/MoS_2_-rGO heterostructure by the wet chemistry approach. The high density of nanoscale interfacial contact between CZTS nanoparticles and MoS_2_-rGO hybrid was demonstrated to favor the photogenerated electron transfer from CZTS directly to MoS_2_ or through rGO, thus reducing the exciton recombination, increasing the activity sites, and enhancing the photocatalytic efficiency for H_2_ generation. The MoS_2_-rGO hybrid has shown a synergistic effect when combined with CZTS, which makes this heterostructure an excellent H_2_ evolution photocatalytic system with a long-term durability.

## Methods

### Synthesis of graphene oxide

Graphene oxide (GO) was synthesized by an improved Hummers’ method^55^. Typically, a mixture of concentrated H_2_SO_4_ (120 mL) and H_3_PO_4_ (13.3 mL) was slowly added to a mixture of graphite flakes (1 g) and KMnO_4_ (6 g), and then heated to 50 °C and stirred for 12 h. After the reaction, the reaction mixture was cooled down to room temperature and poured onto ice (400 mL) with 30% H_2_O_2_ (3 mL). After another 1 h stirring, the mixture was centrifuged at 4,000 rpm for 4 h, and the supernatant was decanted away. The remaining solid material was then washed sequentially with water (200 mL), 37% HCl (200 mL), and ethanol (200 mL). Finally, it was purified by dialysis over one week using a dialysis membrane (Promega SV Minicolumns). The solid obtained was vacuum-dried at 60 °C and the yield is 585 mg.

### Synthesis of MoS_2_

In a typical synthesis of few-layer MoS_2_^44^, 242 mg Na_2_MoO_4_·2H_2_O and 380 mg CS(NH_2_)_2_ were dissolved in 60 mL distilled water. The homogeneous solution was then transferred into a 100 mL Teflon-lined autoclave and held at 210 °C for 24 h. After the completion of reaction, the black precipitate was collected by centrifugation, washed three times with distilled water and ethanol, and then dried in a vacuum oven at 60 °C. The yield of MoS_2_ is 167 mg.

### Synthesis of Cu_2_ZnSnS_4_

CZTS nanoparticles were synthesized by an optimized method described by Chet Steinhagen *et al*.^17^. In a typical procedure, 260 mg Cu(acac)_2_, 145 mg Zn(OAc)_2_, 112 mg SnCl_2_∙2H_2_O, and 15 mL oleylamine were mixed in a three-necked flask at room temperature. After 2 h under vacuum, water and low boiling point solvents in oleylamine were removed by bubbling with N_2_ for 30 min at 120 °C. 65 mg of elemental sulfur was dissolved in 5 mL oleylamine by sonicating for 30 min and then injected into the reaction flask containing metal-oleylamine precursors, and the temperature was raised to 280 °C. After 1 h reaction, the resulting product was cooled down to room temperature. Upon the addition of absolute ethanol, the reaction was quenched and the nanoparticles precipitated out. The obtained nanoparticles were washed three times with ethanol and chloroform in turns. The yield of resulting CZTS nanoparticles is around 180 mg.

### Synthesis of heterostructure

MoS_2_-rGO heterostructure was synthesized by dispersing different amount of GO in the synthesis of MoS_2_^44^. The CZTS/MoS_2_, CZTS/rGO, and CZTS/MoS_2_-rGO heterostructure were prepared by dispersing MoS_2_, rGO, and MoS_2_-rGO with different weight ratio into the sulfur-oleylamine solution, respectively, and injecting it to the metal-oleylamine solution for the growth of CZTS nanoparticles. Platinum nanoparticle decorated CZTS (Pt/CZTS) photocatalyst was prepared by photodeposition method. H_2_PtCl_6_·6H_2_O (10.62 mg) and CZTS (40 mg) were dispersed in 30 mL ethanol by sonication for 1 h and irradiated with 150 W Xe lamp for 30 min. Gold nanoparticle decorated CZTS (Au/CZTS) photocatalyst was prepared by dispersing 6 mg HAuCl_4_·3H_2_O in 5 mL oleylamine at 60 °C. 30 mg of freshly prepared CZTS was dispersed in 3 mL CHCl_3_, which was swiftly injected into the Au-oleylamine precursor solution and the temperature was raised to 90 °C for 10 min.

### Characterization and Measurement

X-ray diffraction spectrum was taken using a SmartLab^®^ X-ray diffractometer (Rigaku) with a diffraction angle 2θ ranging from 10 to 90°. Transmission electron microscopic (TEM) images were obtained using a STEM (JEOL JEM-2100F) operated at 200 kV. The samples were first dispersed in CHCl_3_ by sonication and drop-cast onto holey carbon-coated 400 mesh nickel TEM grids. The elemental compositions of these materials were characterized by an energy dispersive spectrometer (EDS) attached to the STEM. Raman microprobe measurements were performed by a Raman spectrometer (LabRam HR800-UV, Horiba-Jobin Yvon) with excitation wavelength of 532 nm at room temperature. UV–Vis absorption spectra were recorded on a Hewlett Packard Model 8453 Diode Array UV-Vis Spectrophotometer. The sample solutions were prepared in chloroform using a quartz cuvette of 1 cm path length. XPS was performed on an ESCALAB 250Xi ultrahigh vacuum (UHV) surface analysis system (Thermo Fisher Scientific) with a monochromic Al K_α_ X-ray source (1486.6 eV). A thin film was formed by drop-casting the sample solution on single crystal silicon substrate for measurement. Electrochemical measurements were performed using a 1030 A CHI electrochemical station. 5 mg of catalyst and 100 μL of 5 wt% Nafion solution were dispersed in 2 mL of 4:1 v/v water/ethanol by 10 min sonication to form homogeneous suspension. Then, 5 μL of the catalyst ink was drop-cast onto a glassy carbon electrode of 3 mm diameter (surface area: 0.07 cm^2^). A linear sweep voltammogram from 0 to −1.8 V was conducted at a scan rate of 2 mV s^−1^ in a Na_2_S (0.35 M) + Na_2_SO_3_ (0.25 M) solution using a saturated calomel electrode as the reference electrode, a Pt wire as the counter electrode, and a glassy carbon electrode as the working electrode. For photocatalytic hydrogen evolution experiments, catalyst in *ca*. 5 mg was dispersed in an aqueous solution containing 25 mL Na_2_S (0.35 M) + Na_2_SO_3_ (0.25 M), and irradiated with a Newport solar simulator (150 W Xe lamp, ozone free, Air Mass Filter, AM 1.5 Global) of 70 mW cm^−2^ light intensity for 1 h. The amount of generated hydrogen was analyzed by an Agilent 7890B gas chromatograph system equipped with a TCD detector using N_2_ as carrier gas.

## Additional Information

**How to cite this article**: Ha, E. *et al*. Cu_2_ZnSnS_4_/MoS_2_-Reduced Graphene Oxide Heterostructure: Nanoscale Interfacial Contact and Enhanced Photocatalytic Hydrogen Generation. *Sci. Rep.* **7**, 39411; doi: 10.1038/srep39411 (2017).

**Publisher's note:** Springer Nature remains neutral with regard to jurisdictional claims in published maps and institutional affiliations.

## Supplementary Material

Supporting Information
