# Supplementary material for: Cu2ZnSnS4/MoS2-Reduced Graphene Oxide Heterostructure: Nanoscale Interfacial Contact and Enhanced Photocatalytic Hydrogen Generation
Source: Sci Rep. 2017 Jan 3;7:39411. doi: 10.1038/srep39411 (PMC5206717; doi:10.1038/srep39411)
Supplement: Supporting Information [file srep39411-s1.pdf]

Supporting Information

Cu_2ZnSnS_4/MoS_2-Reduced Graphene Oxide Heterostructure: Nanoscale Interfacial Contact and Enhanced Photocatalytic Hydrogen Generation

Enna Ha,[1] Wei Liu,[1] Luyang Wang,[1] Ho-Wing Man,[1] Liangsheng Hu,[1] Shik Chi Edman Tsang,[2] Chris Tsz-Leung Chan,[1] Wai-Ming Kwok,[1] Lawrence Yoon Suk Lee,[1,*] and Kwok-Yin Wong[1,*]

[1]Department of Applied Biology and Chemical Technology and the State Key Laboratory of Chirosciences, The Hong Kong Polytechnic University, Hung Hom, Kowloon, Hong Kong SAR, China.
[2]Wolfson Catalysis Centre, Department of Chemistry, University of Oxford, Oxford, OX1 3QR, UK.

*E-mail: lawrence.ys.lee@polyu.edu.hk; kwok-yin.wong@polyu.edu.hk;

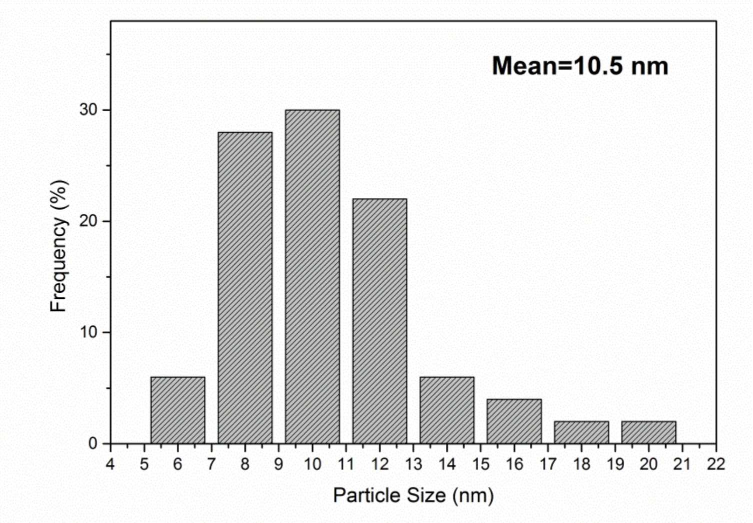

Figure S1 The size distribution histogram of CZTS nanoparticles calculated from TEM images.

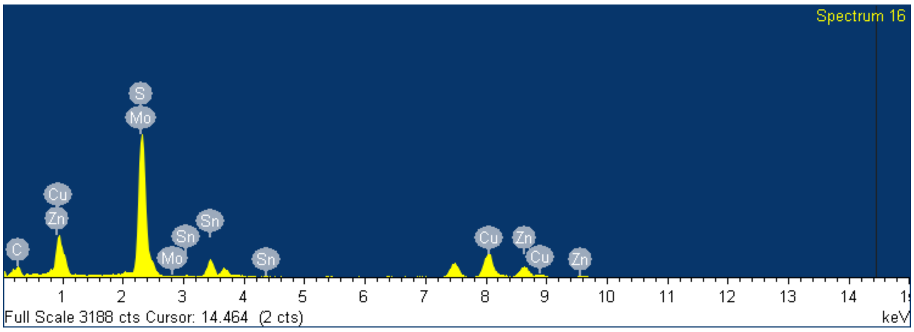

Figure S2 EDS spectrum of CZTS/MoS_2-rGO heterostructure.

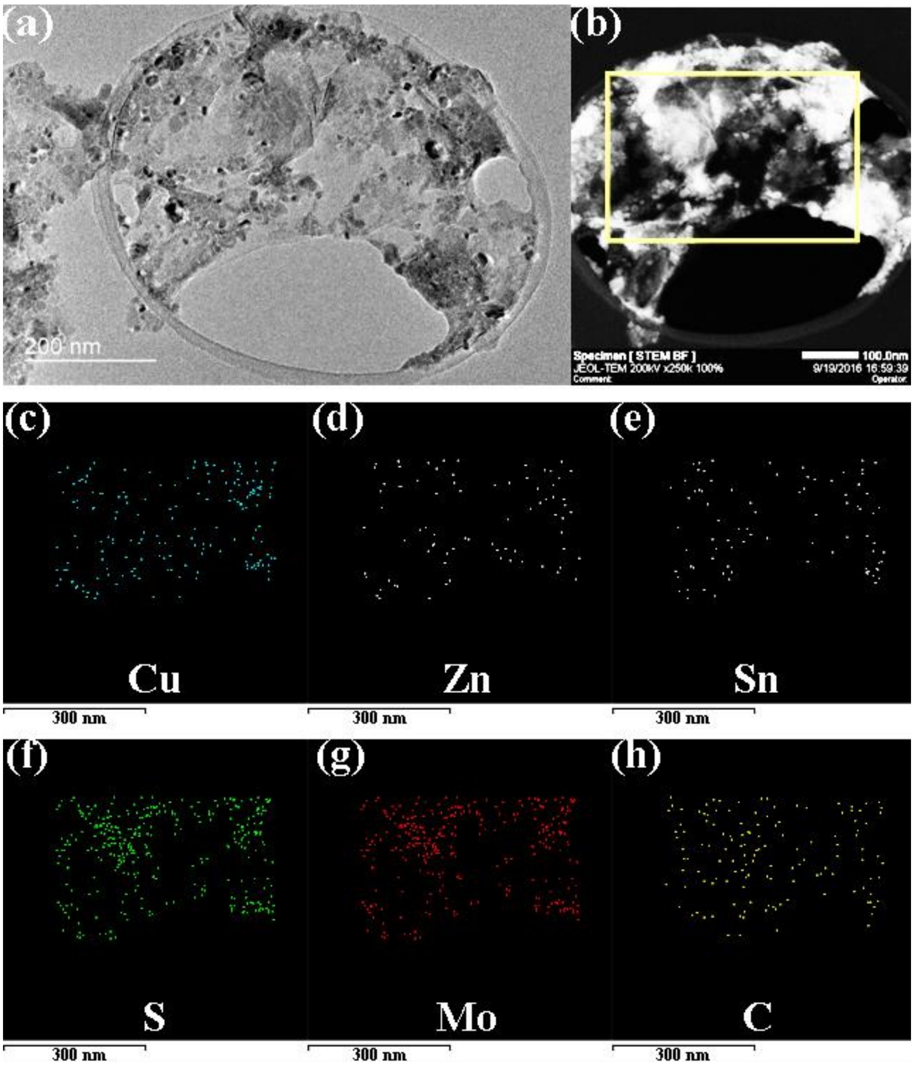

Figure S3 (a) TEM and (b) STEM images of CZTS/MoS_2-rGO and the corresponding EDS elemental mapping images for (c) Cu, (d) Zn, (e) Sn, (f) S, (g) Mo, and (h) C.

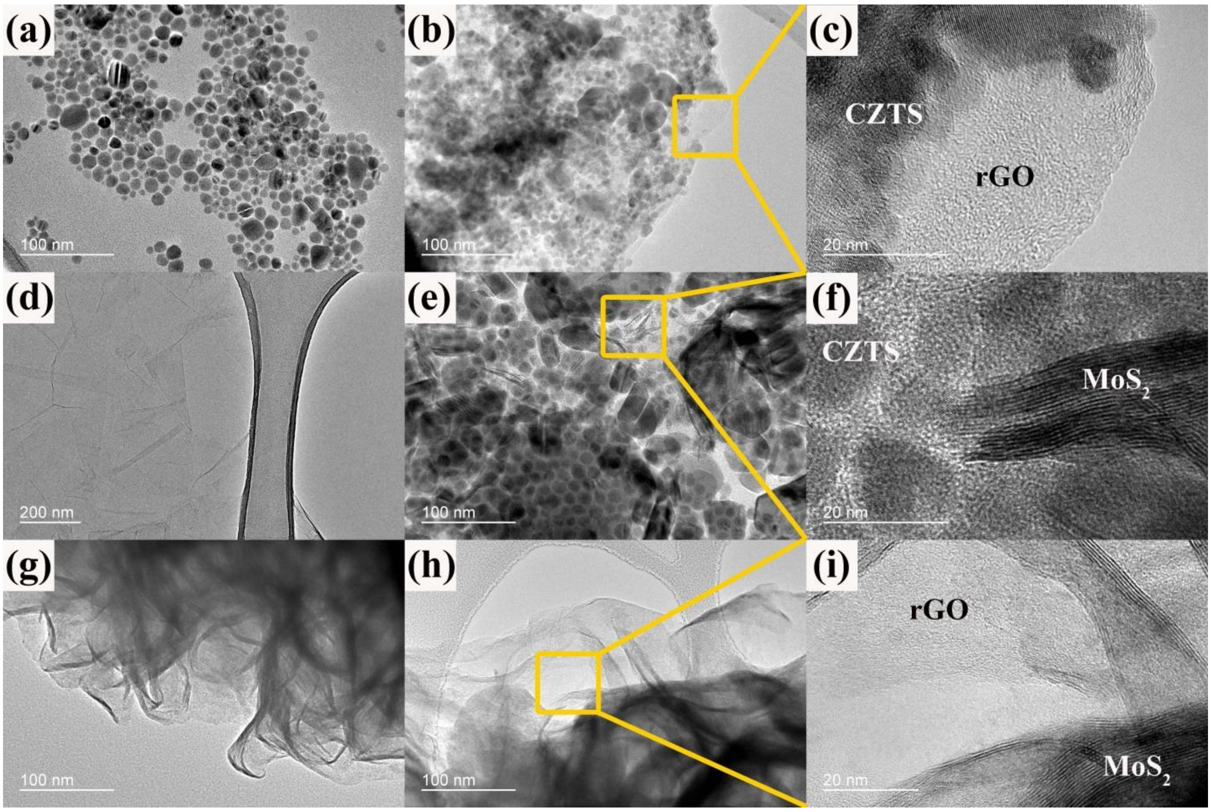

Figure S4 TEM images of (a) CZTS, (b) CZTS/rGO, (d) graphene oxide, (e) CZTS/MoS_2, (g) MoS_2, and (h) MoS_2-rGO. High-resolution TEM images of (c) CZTS/rGO, (f) CZTS/MoS_2, and (i) MoS_2-rGO.

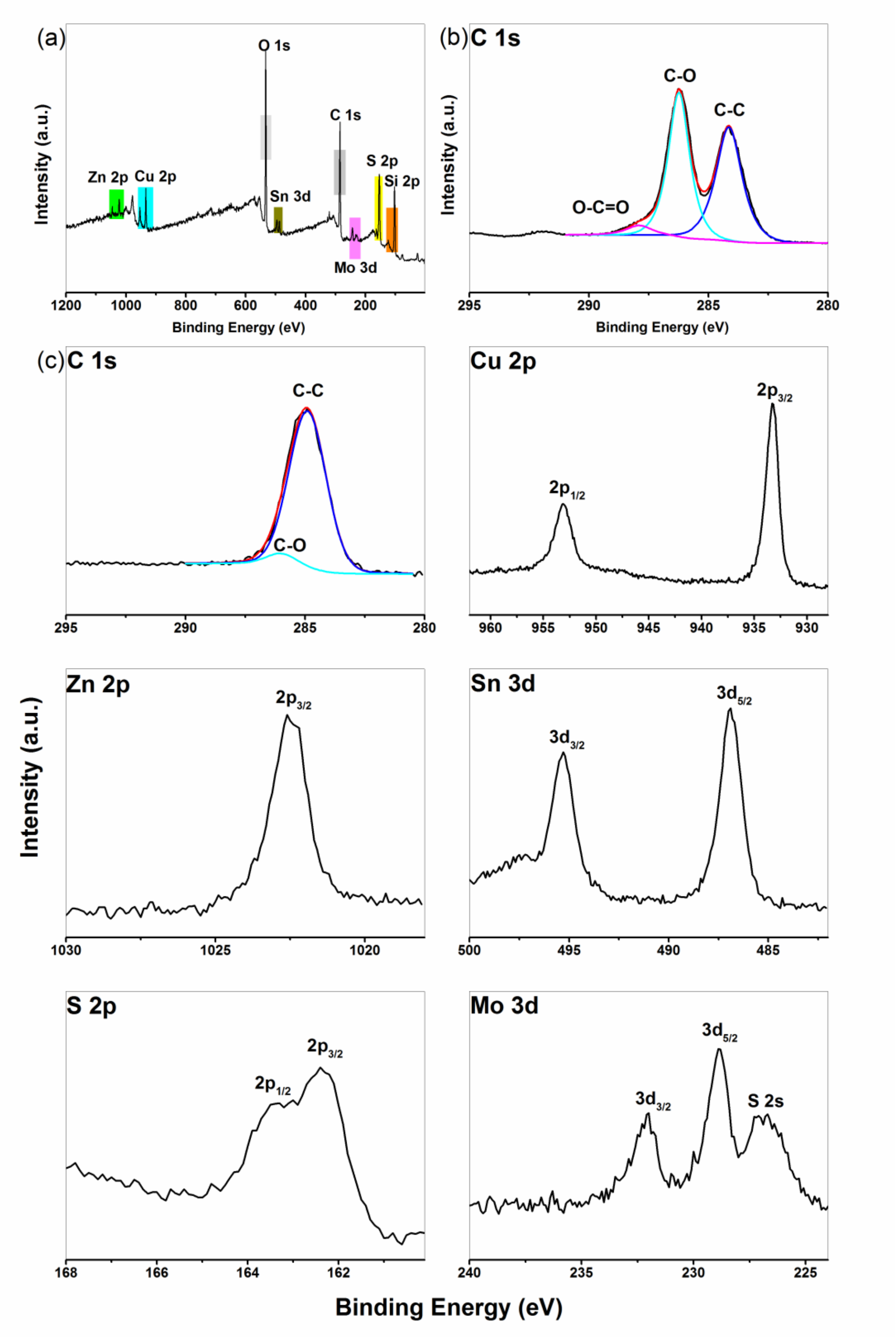

Figure S5 (a) XPS survey spectrum of CZTS/MoS_2-rGO heterostructure. The signal of oxygen and silicon is mainly from the SiO_2 substrate. (b) High-resolution XPS spectrum of C 1s region from graphene oxide and (c) high-resolution XPS analyses for individual elements in CZTS/MoS_2-rGO heterostructure.

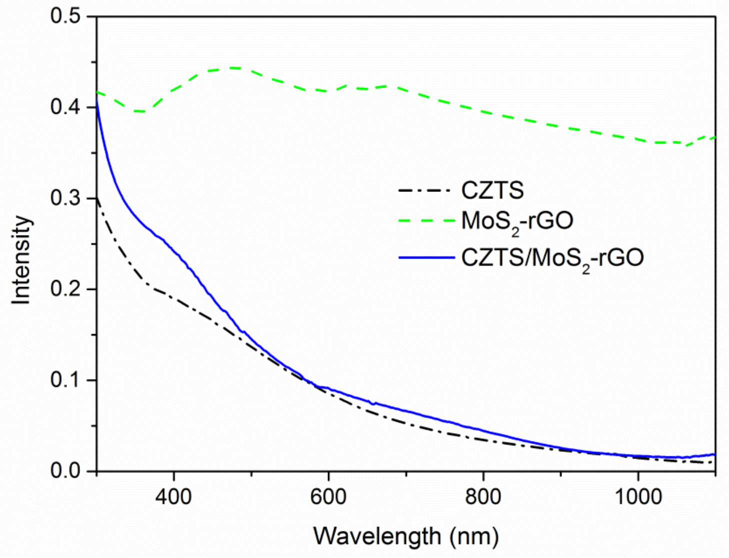

Figure S6 UV-Vis spectra of CZTS, MoS_2-rGO, and CZTS/MoS_2-rGO.

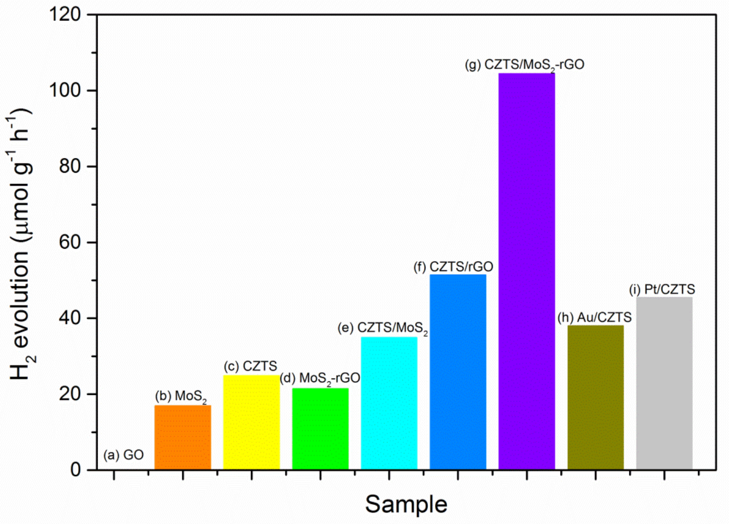

Figure S7 Comparison of the photocatalytic H_2 evolution rates of various photocatalysts. (a) GO, (b) MoS_2, (c) CZTS, (d) MoS_2-rGO, (e) CZTS/MoS_2, (f) CZTS/rGO, (g) CZTS/MoS_2-rGO, (h) Au/CZTS, and (i) Pt/CZTS. Experiment conditions: 1 h irradiation by solar simulator (150 W Xe lamp, ozone free, Air Mass Filter, AM 1.5 Global) of 70 mW cm^{-2} light intensity.

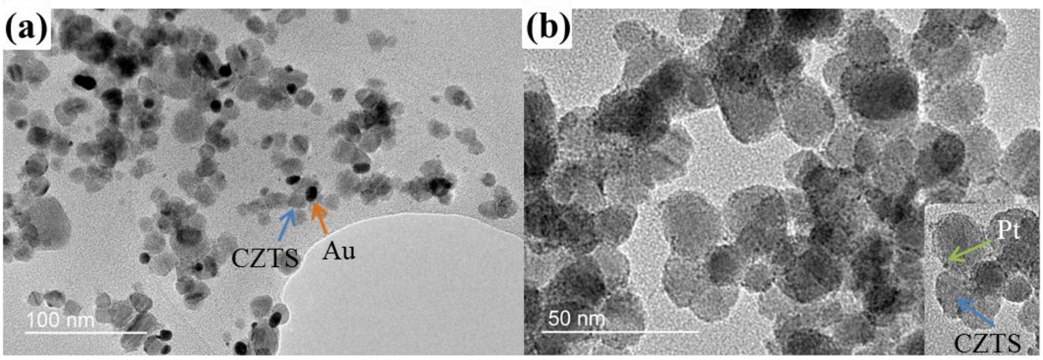

Figure S8 TEM images of (a) Au/CZTS and (b) Pt/CZTS. Inset in (b) shows a high-resolution TEM image of Pt/CZTS.

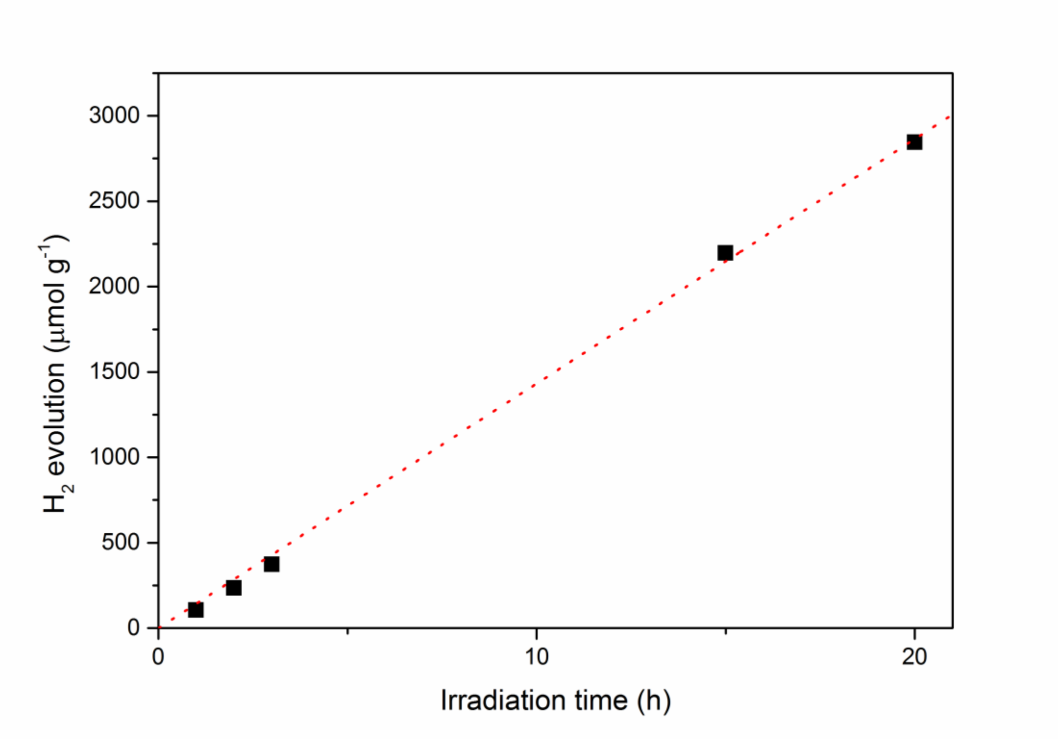

Figure S9 Photocatalytic H_2 evolution by CZTS/MoS_2-rGO heterostructure in a 20 h reaction. The composition of photocatalyst: 90 wt% CZTS, 9 wt% MoS_2, and 1 wt% rGO.

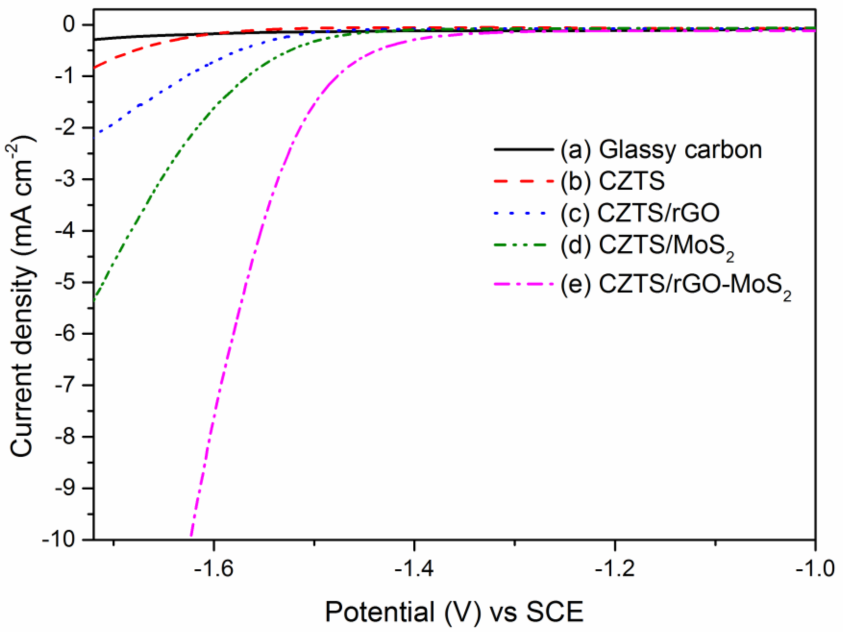

Figure S10 Polarization curves of various samples at a cathodic scan rate of 2 mV s^{-1} in a Na_2S (0.35 M) + Na_2SO_3 (0.25 M) solution: (a) glassy carbon, (b) CZTS, (c) CZTS/rGO, (d) CZTS/MoS_2, and (e) CZTS/MoS_2-rGO heterostructure.
